# Prolonged Pregnancy

**Published:** 1894-03

**Authors:** W. T. Monsarrat

**Affiliations:** Honolulu, H. I.


### PROLONGED PREGNANCY.

BY W. T. MONSARRAT.

Sept. 15th, 1892, a grey mare, nine years old, 16.1 hands, weight about 1,100 to 1,200 pounds, was bred, and as a natural consequence, Aug. 4, 1893, she had a foal. On Sept. 24th, 1893, this foal died, and in the latter part of the month the mare was bred again, and on the 6th Feby., 1894, the mare had another colt, a little over four months from service. The last foal is a large, healthy animal, and is now by its dam. The mare was seen to have both colts by reliable persons, so there is no mistake that the colt belongs to another. I would like to have your views on the subject; by expressing them you will favor myself as well as many interested in this matter.

HONOLULU, H. I.

[Just at the time of the receipt of the above communication of what is evidently a case of prolonged gestation, a bitch was brought to our Infirmary, with a history of having had a litter of puppies four years ago. In perfect health until five weeks ago, when she came in heat and was lined. Since that time has been running down Examination proved the presence of an enormous dead puppy, which has been removed, and the bitch is doing well. —ED.]

